# Non-Invasive Endometrial Cancer Screening through Urinary Fluorescent Metabolome Profile Monitoring and Machine Learning Algorithms

**DOI:** 10.3390/cancers16183155

**Published:** 2024-09-14

**Authors:** Monika Švecová, Katarína Dubayová, Anna Birková, Peter Urdzík, Mária Mareková

**Affiliations:** 1 Department of Medical and Clinical Biochemistry, Faculty of Medicine, Pavol Jozef Šafárik University in Košice, Tr. SNP, 104001 Košice, Slovakia; monika.svecova@upjs.sk (M.Š.); katarina.dubayova@upjs.sk (K.D.); anna.birkova@upjs.sk (A.B.); 2 Department of Gynaecology and Obstetrics, Faculty of Medicine, Pavol Jozef Šafárik University in Košice, Tr. SNP, 104001 Košice, Slovakia; peter.urdzik@upjs.sk

**Keywords:** endometrial cancer, autofluorescence, urine, cancer detection, machine learning

## Abstract

**Simple Summary:**

The incidence of endometrial cancer is increasing, creating a need for fast and efficient diagnostic methods. This study explores a new, non-invasive approach using urinary fluorescence spectroscopy to detect endometrial cancer. By analyzing morning urine samples and utilizing advanced machine learning techniques, we identified prospective spectral markers that differentiate between control, benign, and malignant gynecological patients. Our findings indicate good sensitivity and specificity, with high AUC from machine learning models, suggesting this method could significantly improve early cancer detection. This approach is easier and more affordable, especially in resource-limited settings. It has the potential to change the way endometrial cancer is diagnosed, offering a simpler and more accessible option for patients.

**Abstract:**

Endometrial cancer is becoming increasingly common, highlighting the need for improved diagnostic methods that are both effective and non-invasive. This study investigates the use of urinary fluorescence spectroscopy as a potential diagnostic tool for endometrial cancer. Urine samples were collected from endometrial cancer patients (*n* = 77), patients with benign uterine tumors (*n* = 23), and control gynecological patients attending regular checkups or follow-ups (*n* = 96). These samples were analyzed using synchronous fluorescence spectroscopy to measure the total fluorescent metabolome profile, and specific fluorescence ratios were created to differentiate between control, benign, and malignant samples. These spectral markers demonstrated potential clinical applicability with AUC as high as 80%. Partial Least Squares Discriminant Analysis (PLS-DA) was employed to reduce data dimensionality and enhance class separation. Additionally, machine learning models, including Random Forest (RF), Logistic Regression (LR), Support Vector Machine (SVM), and Stochastic Gradient Descent (SGD), were utilized to distinguish between controls and endometrial cancer patients. PLS-DA achieved an overall accuracy of 79% and an AUC of 90%. These promising results indicate that urinary fluorescence spectroscopy, combined with advanced machine learning models, has the potential to revolutionize endometrial cancer diagnostics, offering a rapid, accurate, and non-invasive alternative to current methods.

## 1. Introduction

Endometrial cancer (EC) is the sixth most common cancer in women worldwide, significantly impacting women’s health [1]. The incidence of EC is increasing due to factors such as advanced age and rising obesity [2]. Early-stage EC is highly treatable, with a five-year survival rate around 80–90% [3]. However, advanced or recurrent EC cases have a poor prognosis, emphasizing the need for improved early detection methods [4]. Current diagnostic methods for EC, including transvaginal ultrasound (TVS), hysteroscopy, and endometrial biopsy, are effective but often very uncomfortable, sometimes painful, and not suitable for routine screenings [5,6].

TVS is commonly used for initial assessments but may lead to further invasive procedures if the results are inconclusive. Hysteroscopy, though providing direct visualization of the endometrial cavity, requires anesthesia and can have many complications. Endometrial biopsy, the gold standard for diagnosis, involves tissue sampling that can be painful and carries several risks, including vasovagal episodes and possible uterine perforation [7].

Given these limitations, there is increasing interest in non-invasive diagnostic techniques for EC [8]. One promising area of research is blood-based biomarkers, which offer a non-invasive way to detect cancer early. Furthermore, circulating miRNAs and circular RNAs in body fluids, such as serum and plasma, have gained attention due to their stability and diagnostic potential. These molecules not only provide molecular insights into cancer progression but also support fertility-sparing treatments in EC patients [9,10]. Researchers are actively working on identifying and validating new panels of serum metabolites using advanced techniques such as mass spectrometry, enabling faster profiling and showing significant potential to enhance diagnostic accuracy over traditional markers such as CA-125. [11].

In addition to blood-based diagnostics, urine-based diagnostics have emerged as an attractive, non-invasive alternative due to the ease of sample collection and the ability of urine to reflect systemic metabolic changes. Urine contains various biomolecules, including metabolites, proteins, and DNA, which can serve as biomarkers for EC [12]. Recent studies have explored urine-derived miRNAs for their potential in non-invasive screening, evaluating them as diagnostic biomarkers for EC and ovarian cancer, with promising results for early detection [13,14]. Metabolomic profiling of urine using techniques such as ultra-performance liquid chromatography mass spectrometry (UPLC-MS) has successfully identified metabolic patterns that distinguish between EC patients and healthy individuals. These metabolic profiles reveal changes in amino acid metabolism and help develop diagnostic models. [15,16].

In addition to metabolomic profiling, techniques such as attenuated total reflection-Fourier transform infrared (ATR-FTIR) spectroscopy have demonstrated high sensitivity and specificity in distinguishing EC patients from healthy individuals, making it a valuable option for screening high-risk populations [17]. Studies utilizing infrared spectroscopy of urine, combined with machine learning, have accurately detected EC by analyzing molecular ‘fingerprints’ present in urine samples [18]. Other vibrational spectroscopic methods, such as mid-infrared absorption and Raman spectroscopy, also offer rapid and cost-effective ways to enhance diagnostic accuracy through the analysis of these molecular signatures [19]. These non-invasive approaches, particularly when combined with machine learning algorithms, present a promising and patient-friendly method for EC detection, complementing traditional invasive diagnostic techniques and improving early detection strategies.

Many metabolites possess native fluorescent properties and are present in biological samples such as tissue, blood, and urine [20]. The metabolism of cancerous tissues differs from that of healthy ones, affecting the composition of natural fluorophores in body fluids [21]. Fluorescence spectroscopy, especially in 3D form, is gaining attention as a non-invasive diagnostic tool for early cancer detection due to its sensitivity and specificity. This technique leverages the autofluorescence properties of endogenous fluorophores in urine, allowing for the detection of cancer-related alterations in a non-destructive, rapid, and cost-effective manner [22]. Compared to traditional diagnostic methods such as mass spectrometry (MS) and nuclear magnetic resonance (NMR), 3D fluorescence spectroscopy is simpler and more affordable, making it well-suited for routine clinical use [23]. It enables the simultaneous detection of multiple metabolites and the monitoring of various metabolic changes without destroying samples. Comparative fluorescence analysis identifies changes in the composition of the analyzed sample even without defining the exact metabolome makeup. Recent advancements in ML have further enhanced the capabilities of 3D fluorescence spectroscopy, improving data analysis and interpretation [24,25,26].

Normal urine contains many native fluorophores, including metabolites of tryptophan, riboflavin, catecholamines, and porphyrins, resulting in strong endogenous fluorescence predominantly in the UV part of the spectrum (due to indole derivatives) [27]. Changes in the expression of these endogenous fluorophores can indicate metabolic disorders related to diseases, including cancer. The use of urinary fluorescence analysis offers the potential to detect urinary metabolites associated with neoplastic processes, potentially providing new directions in the search for predictive and prognostic markers. Previous studies have confirmed the high efficiency of fluorescence spectroscopy as a diagnostic tool for several different cancers, including ovarian, bladder, liver, and malignant melanoma, among others [28,29,30,31].

To the best of our knowledge, the application of urine fluorescence spectroscopy for screening patients with EC and distinguishing them from benign uterine tumors and healthy controls has not been reported. This study pioneers the combination of urinary fluorescence metabolite profiling with machine learning algorithms for early cancer detection, offering a novel approach in cancer diagnostics. By analyzing the unique fluorescence profiles of biofluorophores, this method seeks to provide a reliable, rapid, and patient-friendly diagnostic screening approach that could significantly enhance early detection and improve patient outcomes.

By utilizing the autofluorescence properties of urinary biofluorophores, this technique allows for the detection of cancer-related alterations without the need for invasive procedures. Additionally, the integration of machine learning algorithms improves diagnostic accuracy and opens possibilities for automation, especially in resource-limited settings.

The key contributions of this study are as follows:

- Development of a novel non-invasive diagnostic method
- Improvement in detection sensitivity
- Cost-effectiveness and practicality
- Potential for automation
- Foundation for further research

## 2. Materials and Methods

### 2.1. Study Design

The study group consisted of patients diagnosed with EC (*n* = 77) and benign tumors of the uterus (*n* = 23), along with a control group of gynecological patients (*n* = 96). The control group included 36 women that underwent preventive gynecological examinations and 60 patients who attended follow-up appointments after treatment for gynecological inflammatory diseases. The most frequent diagnoses were inflammatory disease of the cervix uteri (ICD10 code N72, *n* = 24) and other inflammation of the vagina and vulva (N76, *n* = 23). Other diagnoses in this group included dysplasia of the cervix uteri (N87, *n* = 6), female infertility (N97, *n* = 3), salpingitis and oophoritis (N70, *n* = 2), and inflammatory disease of the uterus excluding the cervix (N71, *n* = 2).

The patients with diagnosed EC included 72 with endometrioid carcinoma, 3 with serous endometrial intraepithelial carcinoma, and 2 with mixed EC. Most patients were in the early stages of the disease (Stages I and II, *n* = 53) and had low-grade tumors (*n* = 44). The staging and grading of all gynecological patients (patients with EC and benign patients) was defined based on histopathological examination after the surgery, in accordance with the International Federation of Gynaecology and Obstetrics (FIGO) staging system for endometrial carcinomas and uterine sarcomas [32,33]. This system uses key pathological information such as the extent of myometrial invasion, involvement of the cervical stroma, spread to regional lymph nodes, and the presence of distant metastases to assign specific stages, thereby guiding prognosis and treatment planning.

Endometrial polyps (*n* = 13) and ovarian endometrial cysts (*n* = 7) were the most prevalent types of endometrial lesions in the benign group. Other benign diseases included adenomysosis, leiomyoma, and hematocolpos.

Patients were recruited during their hospitalization or examination at the Gynaecology and Obstetrics Clinic of Louis Pasteur University Hospital in Košice. Written informed consent was obtained from all participants prior to sample collection. The clinical investigations were conducted in accordance with the Declaration of Helsinki, and this study was approved by the Ethics Committee of the Pavol Jozef Šafárik University in Košice, Faculty of Medicine (2024/EK/01011). Detailed age summaries of all groups can be found in Table 1.

### 2.2. Urine Sample Collection, Processing, and Analysis

Urine samples were collected from gynecological patients immediately following their admission or arrival to the hospital before taking any medication or undergoing any procedures. Control group participants provided first morning urine samples after fasting for at least 8 h before sample collection. All urine samples were collected under standardized conditions to ensure consistency and minimize variability. Key urine parameters, including leukocytes, nitrite, pH, specific gravity, protein, glucose, ketones, urobilinogen, bilirubin, and blood, were evaluated semi-quantitatively using a 10-parameter urine strip test (Dekaphan Leuco, Erba Lachema, Brno, Czech Republic).

To ensure sample integrity, samples were immediately centrifuged at 5000 rpm for 10 min at room temperature (Centrifuge Boeco S8, Boeco Germany, Hamburg, Germany) after collection to remove cellular debris. The supernatant was aliquoted into microtubes and stored at −80°C until further analysis. No additional pre-treatment was applied before the dilution and subsequent fluorescence measurements. Before measurement, the urine samples were thawed, and 1 mL of each sample was diluted with deionized water in a 1:3 ratio in a geometric series from the undiluted state to a 1000-fold dilution, ensuring a consistent dilution process for all samples [34].

### 2.3. Instrumentation and Spectral Acquisition

The autofluorescence of urine samples was measured at room temperature using a luminescence spectrophotometer PerkinElmer LS55 (PerkinElmer, Waltham, MA, USA) in a 1 cm quartz cuvette (Helmut Fischer, Sindelfingen, Germany). Each sample was measured using synchronous spectra with a Δλ of 30 nm across a range of 250–550 nm, with a step size of 0.5 nm, excitation/emission slits set to 5/5 nm, and a scan speed of 1200 nm/min. The measurements were performed, and urine total fluorescent metabolome profiles (uTFMP) were constructed as previously described [35]. Birková et al. previously classified seven spectral zones of the uTFMP based on related fluorophores [36]. To ensure consistency in data processing, uTFMPs were standardized using the same parameters for all samples to facilitate comparison between control, benign, and malignant cases.

### 2.4. Statistical and Computational Analysis

For statistical and data analysis, GraphPad Prism 8.0.1 (Boston, MA, USA) and Python (version 3.12) were used. Initially, the normality of the data were assessed using the Shapiro–Wilk test. Given that the data exhibited a non-normal distribution, the Kruskal–Wallis test was used for multiple comparisons, specifically applying Dunn’s multiple comparisons test with Bonferroni correction to control the family-wise error rate [37]. For zone comparisons, Tukey’s multiple comparisons test was applied following one-way ANOVA, with adjustments made for Type I error using the family-wise error rate during pairwise comparisons [38]. Statistical significance was assigned for *p*-values < 0.05, and values of fluorescence intensity were expressed as median ± interquartile range to account for the non-parametric nature of the data.

Following preprocessing, features such as peaks and fluorescence ratios were extracted from the spectra. The Z4a/Z5 and Z6/Z7 ratios were selected based on the progression of the spectra observed in the mean profiles of the uTFMP. To assess the diagnostic potential of the fluorescence ratios, ROC curves were generated using the Wilson/Brown method. This method is particularly effective for calculating confidence intervals in classification models, especially with smaller sample sizes. By applying the Wilson/Brown method, we ensured that the ROC curves more accurately represented the diagnostic performance of the fluorescence data. This approach uses a binomial distribution to calculate confidence intervals, providing more reliable results than traditional methods [39].

The raw fluorescence data were first subjected to smoothing using the Savitzky-Golay filter with a window length of 11 and a polynomial order of 3. This filtering technique was applied to reduce noise while preserving the overall shape and features of the fluorescence spectra, ensuring that any minor fluctuations were smoothed out without distorting the significant peaks. The smoothed data were then used for all subsequent analyses.

To further process the data, uTFMP was transposed by each zone, and the presence of peaks was classified based on the increase in fluorescence units. Specifically, a value of 2 (peak) was assigned if there was an increase of at least 15 units of fluorescence between consecutive measurements within the zone, a value of 1 (slight inclination) for an increase of 5–10 units, and a value of 0 if the increase was less than 5 units.

The dataset was divided into training and testing sets using a 70:30 split ratio. The split was stratified based on the target variable to maintain the proportion of control and malignant samples in both the training and test sets, ensuring that the model was trained and evaluated on representative samples.

Partial Least Squares Discriminant Analysis (PLS-DA) was used to reduce data dimensionality while maximizing the separation between control, benign, and malignant urine samples. [40]. Four machine learning models: Random Forest (RF), Logistic Regression (LR), Support Vector Machine (SVM), and Stochastic Gradient Descent (SGD) were employed to differentiate between controls and patients with EC. These models were implemented and validated using the Scikit-learn library [41]. Each model was built in accordance with the recommendations of the IFCC Working Group on Machine Learning in Laboratory Medicine [42].

To further account for class imbalance and avoid bias, we employed stratified 10-fold cross-validation, ensuring that each fold contained the same class distribution. Each model was also run for 100 repetitions to assess robustness and generalizability. Hyperparameter tuning was performed to improve model performance. For RF, we evaluated the number of trees (100, 150, 200) and tree depths (5, 10, 15), with the optimal configuration being set at 150 trees with a maximum depth of 10. For SVM, the regularization parameter (C) was tested with values of 0.01, 0.1, and 1 and performed best at 1. LR was run with a maximum of 10.00 iterations. For SGD, the loss function was set to logistic regression, and we tested alpha values of 0.000001, 0.0001, and 0.01, with learning rates set to optimal and adaptive. SGD achieved the best performance with an alpha of 0.0001 and the optimal learning rate. These optimal hyperparameters were selected based on model performance during cross-validation to ensure the best predictive accuracy and robustness.

The performance of each model was evaluated based on metrics such as accuracy, sensitivity, specificity, positive predictive value (PPV), negative predictive value (NPV), positive likelihood ratio (PLR), negative likelihood ratio (NLR), and area under the curve (AUC), providing a comprehensive assessment of their diagnostic potential. In addition, confusion matrices were generated for each model to visualize and compare the true positive, true negative, false positive, and false negative rates, offering further insights into classification performance across different data representations.

## 3. Results

### 3.1. Semiquantitative Strip Analysis of Key Urine Parameters

The average pH in the gynecological control group was 6.06. In this group, 5 patients were positive for ketones with a low concentration of 1.5 mmol/L. Among the urine samples, 4 tested positive for leukocytes and 8 for blood, which can indicate inflammation or mild infection, common in gynecological conditions. Additionally, there were occasional positive results for proteins (36 samples) and bilirubin (7 samples). All protein-positive samples were still within the upper range of a physiological state at 300 mg/L. These results correspond to these patients having inflammatory gynecological conditions.

In the benign group, the average pH was 6.43, slightly higher, but still within the normal range. Eight patients were positive for ketones with varying concentrations between 1.5–5 mmol/L, possibly related to metabolic changes due to benign conditions such as fibroids or cysts. Among the urine samples in this group, 8 tested positive for leukocytes, 7 for urobilinogen, 1 for blood, and 3 for hemoglobin. Additionally, there were occasional positive results for proteins (11 samples) and bilirubin (12 samples). Positive bilirubin results might indicate some degree of hemolysis or anemia, which can occasionally be associated with benign gynecological conditions [43].

**Figure 1:**
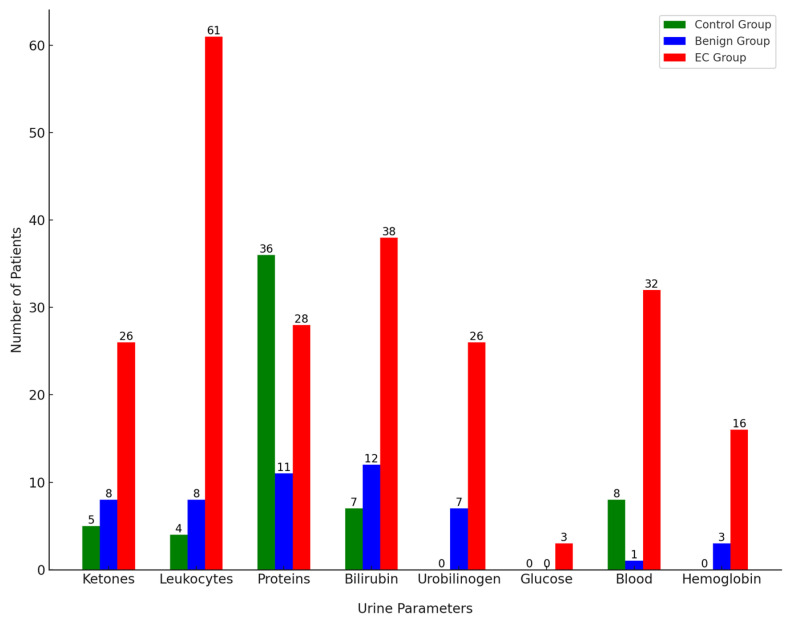
Semiquantitive strip analysis comparison of positive urine parameters.

In the EC group with malignant cases, the average pH was 5.69, indicating a more acidic environment, possibly associated with carcinogenesis and cellular breakdown. Several parameters showed positive results in the urinalysis: 61 samples positive for leukocytes, 28 for proteins, 3 for glucose, 26 for ketones, 26 for urobilinogen, 38 for bilirubin, and 32 for blood components, including 16 specifically positive for hemoglobin. High leukocyte counts (500 mg/dL) indicate significant inflammation or infection. Elevated proteins and the presence of blood components suggest kidney involvement or significant tissue breakdown. The presence of glucose and elevated ketones are more alarming and could indicate metabolic stress or cancer-related cachexia. Figure 1 illustrates the number of patients positive for each urine parameter across the control, benign, and EC groups.

### 3.2. Fluorescent Profiles and Zones

**Figure 2:**
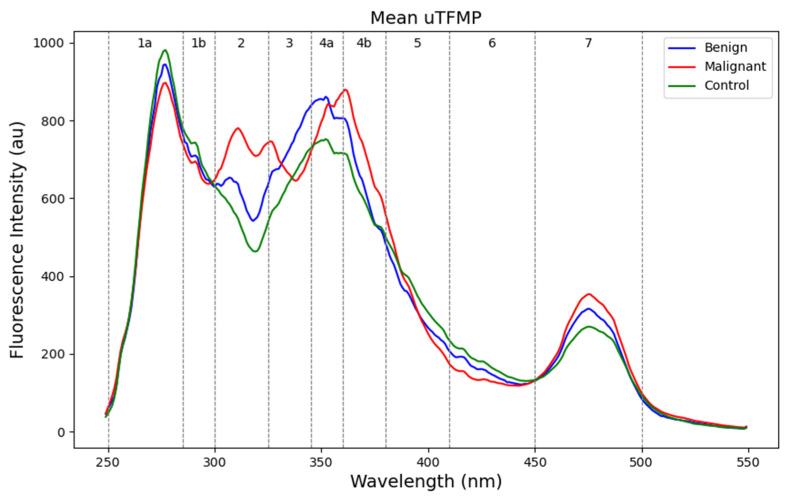
Urinary total fluorescent metabolome profiles (uTFMP) divided into fluorescent zones.

The mean uTFMP curves showed several spectral characteristics distinguishing the different groups (Figure 2). Zones 1a and 1b, characteristic of indole derivatives and catecholamine metabolites (250–300 nm), exhibited the highest fluorescence intensity in the control group and the lowest in the malignant group. This indicates a higher presence or more active metabolism of these compounds in the control group compared to the malignant group. Zone 2 (300–325 nm), containing mainly 5-HIAA (5-hydroxyindoleacetic acid), clearly differentiated the control group from the benign and malignant patients. Zone 3 (325–345 nm) showed a distinct peak for 3-HAA (3-hydroxyanthranilic acid) fluorescence, which was present only in malignant samples. This indicates elevated levels of 3-HAA in malignant samples, confirming the increased catabolism of tryptophan. Zone 4 exhibits fluorescence for various metabolites with similar fluorescent characteristics. Since individual fluorophores have not been identified in this study, we will not specify them. In zones 4a and 4b (345–380 nm), a red shift was present in malignant patients in comparison with the control group. Zone 5 (380–410 nm), characteristic of xanthurenic acid, did not show notable differences among the groups. In Zone 6 (410–450 nm), the fluorescence of xanthopterin and kynurenine was not elevated in benign or malignant samples and had a lower fluorescence intensity overall. Finally, zone 7 (450–500 nm), containing flavins such as FAD and vitamin B_2_, was slightly elevated in malignant samples.

**Figure 3:**
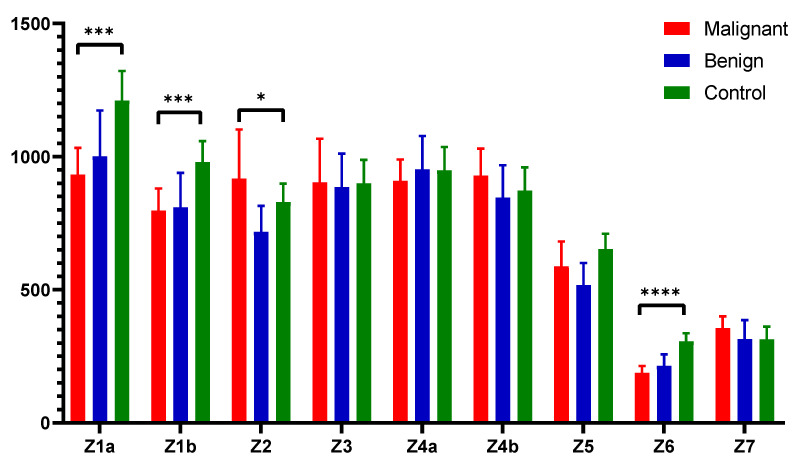
Fluorescent urinary zones. Values are expressed as median ± interquartile range. **** indicates *p* < 0.0001, *** indicates *p* < 0.001, * *p* < 0.05.

Figure 3 illustrates the fluorescent urinary zones and their median ± interquartile range (IQR), highlighting statistical significance between the zones and compared groups. Specifically, zone 1a and zone 1b showed high statistical significance (*p* < 0.001), indicating strong statistical differences. Zone 2 exhibited moderate significance (*p* < 0.05), differentiating the controls from the other groups. Zone 6 demonstrated very strong significance (*p* < 0.0001), underscoring the marked difference between the groups in this spectral zone.

### 3.3. Fluorescent Spectral Markers and Their Clinical Utility

**Figure 4:**
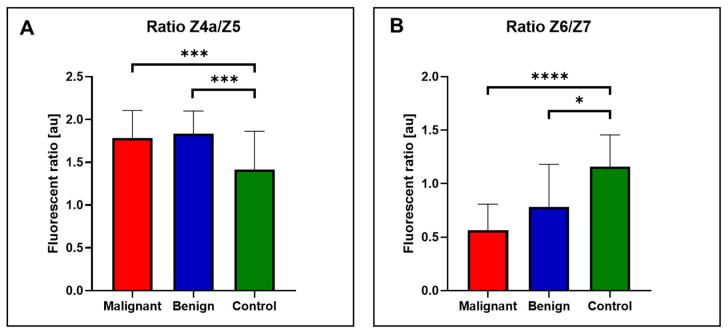
Fluorescent ratios (**A**) Ratio Z4a/Z5. (**B**) Ratio Z6/Z7. Values are expressed as median ± interquartile range. **** indicates *p* < 0.0001, *** indicates *p* < 0.001, * *p* < 0.05.

It was previously established that fluorescence ratios provided greater accuracy than comparing specific selected wavelengths [34]. The Z4a/Z5 ratio was found to be significantly higher in both malignant and benign samples compared to the control group of patients (Figure 4A). This ratio effectively differentiated both EC and benign samples from controls (*p* < 0.001). In contrast, the Z6/Z7 ratio was the lowest in malignant samples, followed by benign samples, and then control samples (Figure 4B). This ratio distinguished malignant samples from control samples with very strong statistical significance (*p* < 0.0001) and also differentiated benign samples from control samples (*p* < 0.05). The Z4a/Z5 and Z6/Z7 ratios define prospective fluorescent spectral markers that help discriminate the EC and benign group of patients from controls.

**Figure 5:**
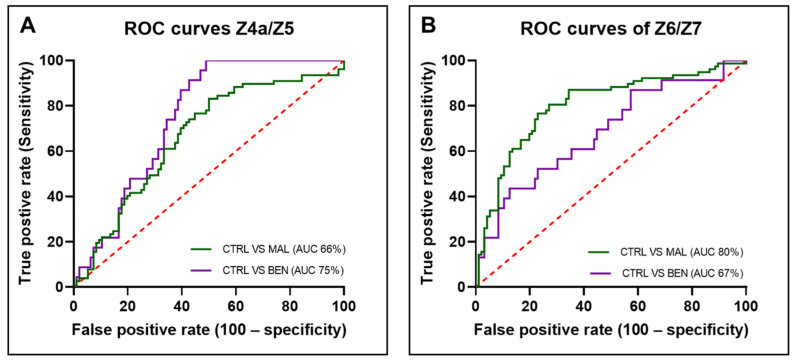
Receiver operating characteristic curves (**A**) Ratio Z4a/Z5 (**B**) Ratio Z6/Z7.

To evaluate the potential clinical utility of these ratios in distinguishing between the groups, ROC curves were plotted (Figure 5). The area under the ROC curve (AUC) for the Z4a/Z5 ratio was 74.91% (STD: 0.04474, 95% CI: 66.14–83.68%, *p* < 0.0001) when comparing controls to benign samples and 66.44% (STD: 0.04184, 95% CI: 58.24–74.64%, *p* < 0.0001) when comparing controls to malignant samples.

For the Z6/Z7 ratio, the AUC was 68.43% (STD: 0.06395, 95% CI: 55.90–80.97%, *p* < 0.001) for controls versus benign samples and 80.34% (STD: 0.03478, 95% CI: 73.53–87.16%, *p* < 0.0001) for controls versus malignant samples. The ROC curves and corresponding AUC values demonstrate that both fluorescence ratios have potential clinical applicability in differentiating between malignant, benign, and control samples. The AUC values, particularly for the Z6/Z7 ratio in malignant samples, indicate a high level of diagnostic accuracy, suggesting these ratios could be useful biomarkers in clinical settings.

### 3.4. Classification and Machine Learning Models

**Figure 6:**
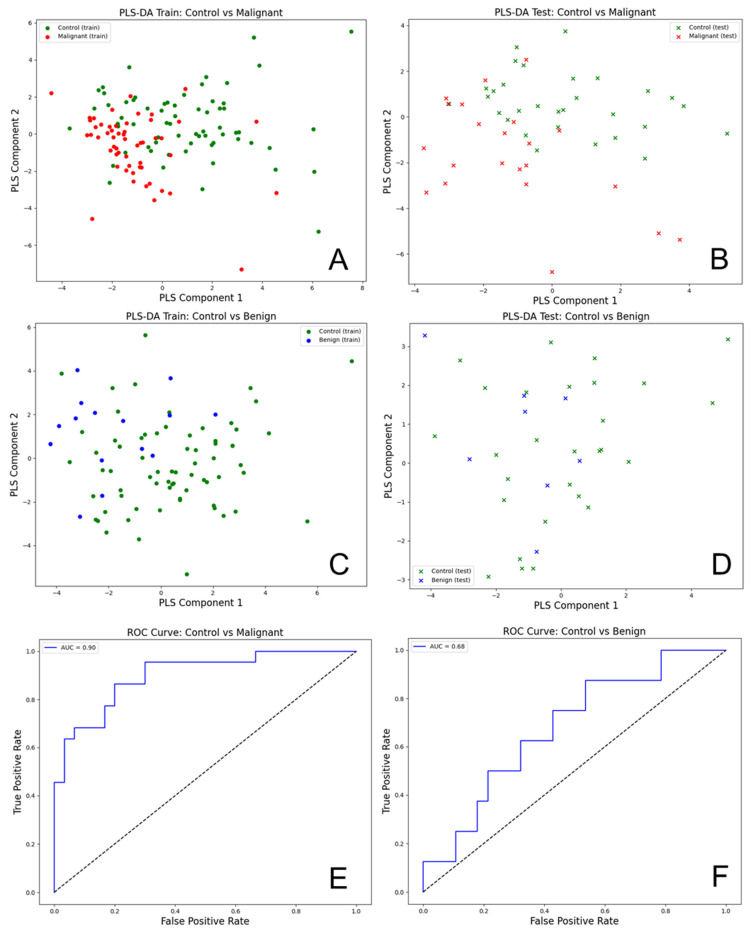
Partial Least Squares Discriminant Analysis (PLS-DA) (**A**) Train set between controls and malignant samples; (**B**) Test set between controls and malignant samples; (**C**) Train set between controls and benign samples; (**D**) Test set between controls and malignant samples; (**E**) ROC curve between controls and malignant samples; (**F**) ROC curve between controls and benign samples.

The Partial Least Squares Discriminant Analysis (PLS-DA) was performed on the transposed data of the urinary total fluorescence metabolite profile (uTFMP) and created fluorescent ratios (Figure 6).

The R^2^ value of 0.824 indicates that the model explains about 82.4% of the variance in the response variable, which signifies a good fit. Additionally, the Q^2^ value of 0.823 demonstrates that the model has strong predictive power and is likely to perform well on new, unseen data. This indicates that the model generalizes well beyond the training data.

The analysis of urine spectra for EC versus controls using PLS-DA demonstrated a high level of accuracy and effectiveness in differentiating these groups. For the PLS-DA model comparing control and malignant samples, the following performance metrics were obtained: For control samples, the precision was 0.91, recall was 0.70, and F1-score was 0.79, with support of 30 samples. For malignant samples, the precision was 0.69, recall was 0.91, and F1-score was 0.78, with a support of 22 samples. Overall, the model achieved an accuracy of 0.79, with a macro average precision of 0.80, macro average recall of 0.80, macro average F1-score of 0.79, weighted average precision of 0.82, weighted average recall of 0.79, and weighted average F1-score of 0.79. The AUC for the ROC analysis was 0.90, indicating excellent discriminatory power between the control and malignant groups.

For the model comparing control and benign samples, the performance metrics were as follows: precision of 0.79, recall of 0.96, and F1-score of 0.87 for control samples (support: 28). For benign samples, the precision was 0.50, recall was 0.12, and F1-score was 0.20 (support: 8). The overall accuracy was 0.78, with a macro average precision of 0.65, recall of 0.54, and F1-score of 0.54, as well as a weighted average precision of 0.73, recall of 0.78, and F1-score of 0.72. The AUC for the ROC analysis was 0.68, indicating moderate discriminatory power. The PLS-DA scatter plots for the training and test datasets showed some overlap between control (green) and benign (blue) samples, highlighting the challenge in distinguishing benign samples from controls.

To evaluate the diagnostic potential of this method, several classification machine learning models were constructed to distinguish EC from the control group of gynecological patients using two types of data representations: transposed fluorescent zones and the whole urine profile (uTFMP).

For the transposed fluorescent zones, the highest performance was demonstrated by the Support Vector Machine (SVM), with an accuracy of 0.77 and an AUC of 0.87. Sensitivity (0.73) and specificity (0.79) were balanced by this model, yielding a positive predictive value (PPV) of 0.74 and a negative predictive value (NPV) of 0.79. The positive likelihood ratio (PLR) was 4.07, and the negative likelihood ratio (NLR) was 0.34. Random Forest (RF) followed closely, with an accuracy of 0.78 and an AUC of 0.86. Logistic Regression (LR) also performed well, with an accuracy of 0.79 and an AUC of 0.85. Slightly lower metrics were observed for Stochastic Gradient Descent (SGD), with an accuracy of 0.78 and an AUC of 0.81.

When using the uTFMP, LR and SVM outperformed the other models. LR achieved an accuracy of 0.83 and an AUC of 0.90, while SVM also reached a high performance with an accuracy of 0.81 and an AUC of 0.90. RF showed strong performance with an accuracy of 0.77 and an AUC of 0.85, and SGD performed well with an accuracy of 0.80 and an AUC of 0.83.

Overall, LR and SVM were the top-performing algorithms across both data representations, demonstrating strong and consistent results. LR particularly excelled in terms of both accuracy and AUC when using the uTFMP data. SVM also provided excellent performance, particularly with the transposed fluorescent zone representation. RF and SGD, while slightly behind LR and SVM, still offered robust results, indicating their potential utility in this diagnostic context. Detailed performance metrics of the machine learning models are summarized in Table 2.

**Figure 7:**
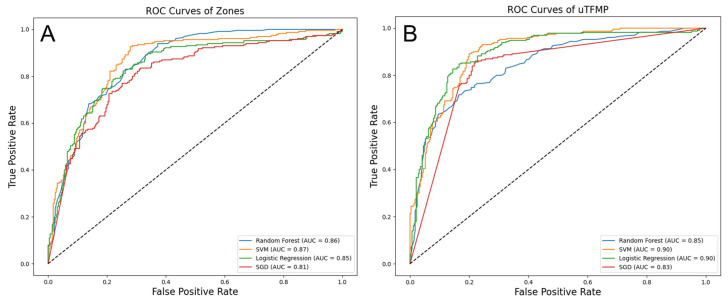
ROC curves of built machine learning models (**A**) ML based on fluorescent zones and spectral ratios (**B**) ML based overall urinary total fluorescent metabolome profile.

The ROC curves illustrate the trade-offs between sensitivity and specificity for each classifier (Figure 7). In the transposed fluorescent zone representation, SVM demonstrated the highest AUC of 0.87, indicating the best overall performance in distinguishing EC from the control group. RF followed with an AUC of 0.86, showing strong performance as well. LR had an AUC of 0.85, and SGD had the lowest AUC of 0.81 among the models evaluated. In the uTFMP representation, both LR and SVM achieved the highest AUC of 0.90, showcasing their superior diagnostic potential. RF and SGD had AUCs of 0.85 and 0.83, respectively. These AUC values further corroborate the effectiveness of LR and SVM in accurately identifying EC cases, making them the most reliable models in this study.

**Figure 8:**
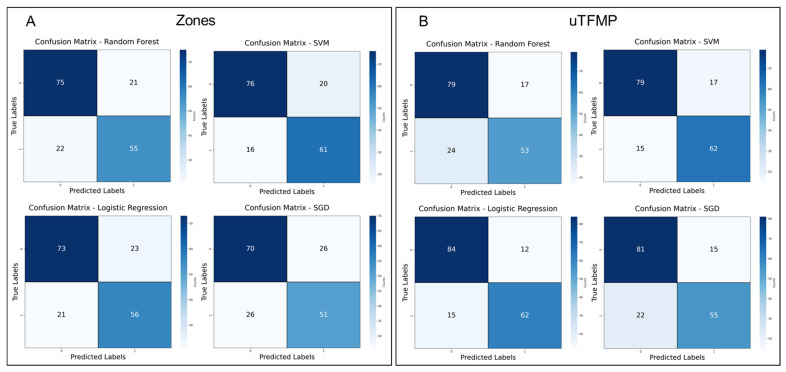
Confusion matrices for machine learning models: (**A**) fluorescent zones and spectral ratios. (**B**) overall urine total fluorescent metabolome profiles.

To further evaluate the diagnostic potential of the machine learning models developed to distinguish between EC patients and the control group, confusion matrices were created for both the transposed fluorescent zones and spectral ratios as well as the overall uTFMP, as shown in Figure 8.

In the transposed data models (Figure 8A), SVM slightly outperformed RF, with 16 false negatives and 20 false positives for SVM, compared to 22 false negatives and 21 false positives for RF. Although both models demonstrated strong classification ability, SVM’s lower misclassification rates indicated a subtle improvement in performance. Logistic Regression (LR) also performed comparably, with 21 false negatives and 23 false positives. However, Stochastic Gradient Descent (SGD) showed the poorest performance, with 26 false negatives and 26 false positives, indicating its lower sensitivity and specificity compared to the other models in this data representation.

For the ML models built on the overall uTFMP data (Figure 8B), RF continued to perform well, with 24 false negatives and 17 false positives, reflecting its ability to distinguish EC patients from controls effectively. SVM performed similarly, with 15 false negatives and 17 false positives, maintaining consistent effectiveness across different data representations. LR exhibited the best performance, with only 15 false negatives and 12 false positives. However, SGD, while still demonstrating some diagnostic potential, continued to underperform relative to the other models, with 22 false negatives and 15 false positives.

The key results:

- LR and SVM showed the best overall performance
- LR particularly excelled with the uTFMP data, while SVM performed well across both data sets
- RF performed well but had higher misclassification rates
- SGD showed lower performance but still demonstrated diagnostic potential

## 4. Discussion

Early and accurate detection of endometrial cancer (EC) is crucial for improving patient outcomes, especially given the rising incidence globally. Traditional diagnostic methods, while effective, often involve invasive procedures and high costs, which can be a barrier to early-stage screening. Therefore, there is a significant need for fast, reliable, and affordable screening tools. Comparative 3D fluorescence analysis of urine offers a promising non-invasive alternative that could facilitate early detection and monitoring of EC and potentially other cancers.

Urine, as a non-invasively obtained biological material, has great diagnostic potential. It contains a wealth of diagnostic information but is an analytically challenging biological system due to its concentration variability and the influence of diet on its composition. However, the problem of concentration variability was eliminated by the introduction of the urinary total fluorescent metabolome profile (uTFMP) [35].

This study aimed to observe and study the autofluorescence of urine samples as a screening tool for differentiating patients with EC, or benign tumors of the uterus, from a control group of gynecological patients. Collecting controls with the same age distribution as the EC patient group is challenging because older women, who are more likely to develop EC, often do not attend gynecological checkups. Furthermore, those who do attend are rarely considered healthy, complicating the recruitment of age-matched controls.

The uTFMP was utilized to distinguish between these groups, revealing several distinct spectral characteristics. In zones 1a and 1b, indicative of indole derivatives and catecholamine metabolites, the highest fluorescence intensity was observed in the control group, while the malignant group showed the lowest intensity. This suggests a higher presence or more active metabolism of these compounds in the control group, potentially due to inflammatory processes such as bacterial infections in the urogenital tract. These findings are consistent with previous research that links metabolic changes in inflammatory conditions to varying fluorescence intensities [44]. Zone 2, containing mainly 5-HIAA, clearly differentiated the control group from the benign and EC patients. This finding aligns with previous work where a similar trend was observed in this spectral range in the study of malignant melanoma [31]. In zone 3, which exhibited fluorescence from 3-HAA, malignant samples showed elevated levels, confirming the increased catabolism of tryptophan. This elevation in tryptophan catabolites aligns with findings from various cancer studies, highlighting that such metabolic shifts are a common feature in malignancy [29,31,45,46]. Our study found a possible NADH red shift and elevated FAD levels in malignant urine samples, indicating altered metabolic processes and changes in redox state associated with cancer. These findings align with reports of altered NADH fluorescence in melanoma [47] and cancer detection studies highlighting NADH and FAD as potential spectral biomarkers [21,45,48]. Interestingly, zone 6 was not elevated in benign or malignant samples but had lower fluorescence overall. This contrasts with elevated levels observed in ovarian cancer patients [46], suggesting variability based on cancer type or patient population in EC.

Comparison of the average fluorescence profiles of the different groups is not ideal due to the inherent variability in fluorescence profiles among individuals. This variability can obscure significant differences and lead to misleading conclusions when only average values are considered. Instead, the ratios of selected fluorescence zones provide a more robust and reliable predictive value. These ratios define so-called fluorescent spectral markers and can highlight specific metabolic shifts and abnormalities that are more consistent across different individuals within the same group, offering a clearer distinction between control, benign, and malignant samples.

The Z4a/Z5 ratio, representing the balance between NADH and xanthurenic acid, provided good differentiation between control and malignant samples. NADH plays a crucial role in cellular respiration, and its levels are often altered in cancer cells due to metabolic reprogramming, including the Warburg effect [49]. Xanthurenic acid, a tryptophan metabolite, has also been associated with oxidative stress and immune modulation, processes that are disrupted in malignancy [50]. Similarly, the Z6/Z7 ratio, comparing kynurenine and FAD, showed strong diagnostic potential. Kynurenine, a key metabolite in tryptophan catabolism, is known to be elevated in various cancers, including EC, contributing to immune suppression and tumor progression [51,52]. Elevated FAD levels, which reflect alterations in cellular redox states and energy metabolism, are characteristic of cancer cells [53].

Both the Z4a/Z5 and Z6/Z7 ratios exhibit potential as prospective spectral biomarkers for distinguishing between benign gynecological samples or EC samples from a control group, as evidenced by their respective ROC AUC values. The highest AUC, 80.34% for the Z6/Z7 ratio in malignant samples, indicates strong diagnostic accuracy, despite the lower AUC values potentially influenced by unequal data sets. In comparison with published studies utilizing fluorescence ratios of urine, our results align, demonstrating the clinical applicability of these ratios as reliable biomarkers [21,48]. Although these studies focused on multiple ratios and observed variability in their performance, our findings fall within the acceptable range, reinforcing the potential of these spectral markers for non-invasive cancer detection.

Our results compare favorably with commonly used blood biomarkers, such as HE4 (human epididymis protein 4) and CA125, which are often used in clinical settings for the diagnosis of endometrial and ovarian cancers. While HE4 and CA125 are established markers, their diagnostic performance in EC detection has limitations. Studies have reported AUC values ranging between 70–85% for HE4 and between 65–80% for CA125, depending on the cohort and the stage of the disease [54]. In comparison, our Z6/Z7 ratio demonstrates a comparable AUC of 80.34%, suggesting similar diagnostic accuracy but with a simpler, non-invasive urine-based approach. Unlike blood biomarker tests, urine diagnostics are pain-free, more convenient for patients, and provide an opportunity for more frequent testing, thus offering a better patient experience and improved accessibility in clinical practice.

In comparison to other non-invasive biomarkers such as urinary miRNAs, our Z6/Z7 ratio shows similar diagnostic potential. Studies have demonstrated that, for example, miR-92a and other miRNAs can achieve AUC values between 75–85% for distinguishing EC from healthy controls [55]. While both approaches offer the benefits of non-invasive testing, the Z6/Z7 ratio provides a simpler and more accessible method. Unlike miRNA detection, which requires more complex molecular testing, our technique is easier to implement in clinical practice, potentially allowing for more frequent, patient-friendly testing and broader clinical application.

To further validate the diagnostic potential of this method, several machine learning models were constructed. The Partial Least Squares Discriminant Analysis (PLS-DA) showed high accuracy in differentiating EC from control samples. However, the model demonstrated moderate performance in distinguishing benign samples from control samples. The disparity in sample sizes (96 control, 77 malignant, and 23 benign) may contribute to the model’s limited effectiveness in identifying benign samples. In comparison, Shao et al. used PLS-DA on UPLC-Q-TOF/MS data for urine metabolomic profiling in endometrial cancer detection, achieving high diagnostic performance, albeit with a more expensive technique [56]. Meza Ramirez et al. employed infrared spectroscopy combined with Orthogonal Projection to Latent Structures (OPLS) as a pre-processing technique before applying PLS-DA and optimizing their results [18]. Despite using different methodologies, both studies support our findings by demonstrating the effectiveness of PLS-DA in identifying EC from urine samples. These results highlight the potential of the PLS-DA model for diagnostic applications in non-invasive EC detection, particularly for differentiating malignant samples from controls.

While previously mentioned studies using spectroscopic methods such as infrared spectroscopy and ambient mass spectrometry have shown high diagnostic accuracy, our approach using fluorescence spectroscopy offers a more rapid and cost-effective alternative for large-scale screening. The SVM model using transposed fluorescent zones demonstrated the highest performance with an AUC of 0.87. When using the whole uTFMP, both LR and SVM achieved the highest AUC of 0.90, showcasing their superior diagnostic potential. These results are comparable to a previous study, which achieved an accuracy of 0.89 using SVM on metabolic data, although their method is significantly more expensive [56]. Overall, our results demonstrate the efficacy and practicality of fluorescence spectroscopy combined with SVM and LR for non-invasive EC detection.

To assess the diagnostic potential of our machine learning models, confusion matrices were generated for both the transposed fluorescent zones and the overall urine profile. These matrices revealed LR and SVM as the top-performing models, demonstrating strong accuracy in distinguishing EC patients from controls. Although false positives are less concerning in a screening context due to follow-up tests such as imaging or biopsy, reducing them is still important to lower patient anxiety and healthcare costs [57]. False negatives, where cases of EC might be missed, pose a greater risk due to potential treatment delays, though they would likely be identified in later diagnostic stages [58]. Mitigation strategies, such as increasing the sample size, especially for benign and control cases, and improving the models, will help minimize both false positives and false negatives. Additionally, integrating this tool into a multi-modal diagnostic framework, commonly seen in other machine learning studies that combine traditional diagnostic methods such as blood biomarkers and imaging techniques with ML algorithms, can significantly enhance the overall accuracy and performance of the model [54,59].

Ultimately, the combination of high diagnostic accuracy, non-invasiveness, and cost-effectiveness makes our method, particularly with LR and SVM, a promising tool for early-stage EC detection. Future work should focus on balancing sensitivity and specificity, ensuring the tool is optimized for clinical use as an efficient and accessible screening method. As this method is intended to be a quick and affordable screening tool, its primary goal is to identify patients who require further diagnostic evaluation rather than providing a definitive diagnosis. With these refinements, the tool is well-positioned to support early cancer detection, guiding healthcare professionals toward more precise, invasive diagnostic procedures when necessary.

To provide additional context for the performance of our method, Table 3 presents a comparison with other state-of-the-art diagnostic techniques for EC. Our approach, leveraging urinary fluorescence spectroscopy combined with ML, achieves a competitive AUC of 0.90. This method also offers significant advantages, including its non-invasive nature and ease of implementation, making it a promising option for clinical applications. In comparison, Meza Ramirez et al. achieved a higher AUC of 0.99 using infrared spectroscopy, but their method required urine collection via catheter, making it a more invasive sampling approach, which could limit its broader clinical adoption [18]. Bouziyane et al. reported an AUC of 0.93 using RT-qPCR for miR-21 in tissue samples, but the need for tissue biopsies makes it more invasive than our urine-based method [60]. Njoku et al. (2022) demonstrated a range of AUC values, with urine CA125 alone yielding an AUC of 0.89 and HE4 an AUC of 0.69. However, when combined with transvaginal ultrasound-measured endometrial thickness, the diagnostic model achieved an AUC of 0.96, offering a non-invasive yet more complex and expensive approach [59]. Finally, Barr et al. (2022) reported an AUC of 0.77 from blood biomarkers (CA125 and HE4). Their results also varied, with the combined model including BMI and parity achieving an AUC of 0.91 in premenopausal women [54]. Although they demonstrated diagnostic potential, the use of blood biomarkers highlights the limitations of traditional testing in terms of invasiveness and diagnostic accuracy. While some methods demonstrate slightly higher diagnostic performance, our study strikes a balance between accuracy, simplicity, and patient comfort, positioning it as a strong alternative for widespread, non-invasive screening of EC.

A key challenge in deploying ML models in healthcare is ensuring that clinicians understand and trust the models’ predictions. Explainable AI (XAI) methods offer a solution by providing insights into how models reach their conclusions, thereby improving both model performance and trustworthiness in clinical settings [61,62]. XAI allows clinicians to verify the model’s decisions against established clinical literature, ensuring that the AI identifies biologically relevant patterns [62,63]. This capability is particularly important when using fluorescence profiles, as XAI can help elucidate which spectral features or zones contribute most to distinguishing between benign, malignant, and control samples [61,63].

Additionally, XAI supports ethical and legal frameworks by addressing transparency issues, such as the “right to explanation” under the European Union’s GDPR [64], fostering trust in AI systems. This transparency is crucial for encouraging clinician acceptance and trust, as they need confidence that AI systems are safe, interpretable, and aligned with clinical protocols. Furthermore, explainability can help ensure ethical decision-making, especially in high-stakes applications such as healthcare [65].

As research progresses, integrating XAI with urinary fluorescence profiling can significantly enhance diagnostic precision while also adhering to the ethical standards expected in medical applications [64,65]. The future development of this study should further explore the potential of XAI to explain model results and verify them with clinical literature, which will be crucial for gaining acceptance and trust from healthcare stakeholders [61,63].

Our study’s findings suggest that urinary fluorescence profiles, combined with ML models, can effectively distinguish between control, benign, and malignant samples, offering prospective diagnostic markers for early-stage EC. The non-invasive nature of this technique, coupled with its cost-effectiveness, makes it a promising tool for large-scale screening and early detection.

Integrating this method into current diagnostic workflows would provide a preliminary screening tool that could guide further diagnostic procedures, such as imaging or biopsy, especially for patients identified as high-risk. This approach could reduce the need for invasive tests in low-risk patients while ensuring more targeted and timely interventions for high-risk individuals. However, its clinical implementation may face barriers, including the need for larger validation cohorts and the establishment of infrastructure for fluorescence-based screening in clinical settings.

While the preliminary results are promising, further validation with larger cohorts is necessary to confirm these findings. Expanding the analysis to include more benign cases and exploring other cancer types could increase the generalizability and applicability of this affordable diagnostic approach. Overcoming these barriers will be essential to successfully integrating this method into clinical workflows, ensuring it provides a cost-effective, accurate, and scalable solution for early cancer detection.

## 5. Conclusions

In conclusion, this study provides compelling evidence for using urinary fluorescence spectroscopy as a screening tool for endometrial cancer. The method’s good sensitivity and specificity, non-invasive nature, and cost-effectiveness make it a valuable addition to current diagnostic techniques. Machine learning models further enhance its accuracy, making it a robust tool for clinical applications, especially in resource-limited settings. As this is a pilot study, further research is essential to validate these findings. Larger cohorts, including more patients in the benign group and healthy volunteers, are needed to ensure reliability and generalizability. This study’s limitations, such as the variability in fluorescence profiles among individuals, must be acknowledged. Despite these challenges, the advantages of this approach over traditional diagnostic methods are significant. Traditional diagnostics often involve invasive procedures and high costs, which can hinder early detection. Compared to other spectroscopic methods or traditional metabolomics, urinary fluorescence spectroscopy offers a more practical alternative due to its ease of use and lower cost. Additionally, its non-invasive nature makes it accessible to a broader population regardless of age. Future studies should aim to reproduce these results in larger, more diverse populations and explore the technology’s potential to differentiate various molecular profiles of endometrial cancer. Expanding research to include other gynecological cancer types could further enhance the diagnostic approach’s generalizability. In summary, while further validation is necessary, this study lays the groundwork for a promising new diagnostic tool that could significantly impact the early detection and management of endometrial cancer, ultimately improving patient outcomes.

## Figures and Tables

**Table 1:** Patient age distribution summary.

	N	Age in Years		
		Minimum	Maximum	Mean ± SD
Controls	96	18	65	36.4 ± 11.66
Benign patients	23	21	46	32.7 ± 6.25
EC patients	77	30	80	60.9 ± 11.5
EC Stage I	35	30	77	55.5 ± 12.3
EC Stage II	19	46	72	60.1 ± 9.5
EC Stage III	10	50	77	70.1 ± 8.6
EC Stage IV	9	51	80	64.0 ± 10.1
EC Stage V	4	58	65	61.5 ± 4.9

**Table 2:** Performance of ML algorithms differentiating between EC and controls.

Zones	Sensitivity	Specificity	PPV	NPV	PLR	NLR	Accuracy	AUC
RF	0.71	0.83	0.77	0.79	4.88	0.35	0.78	0.86
SVM	0.73	0.79	0.74	0.79	4.07	0.34	0.77	0.87
LR	0.81	0.77	0.74	0.84	3.66	0.24	0.79	0.85
SGD	0.77	0.79	0.75	0.82	3.92	0.29	0.78	0.81
**uTFMP**	**Sensitivity**	**Specificity**	**PPV**	**NPV**	**PLR**	**NLR**	**Accuracy**	**AUC**
RF	0.67	0.86	0.78	0.77	4.17	0.43	0.77	0.85
SVM	0.78	0.83	0.79	0.83	5.30	0.26	0.81	0.90
LR	0.78	0.87	0.83	0.84	4.97	0.23	0.83	0.90
SGD	0.76	0.83	0.80	0.82	5.54	0.29	0.80	0.83

PPV, Positive predictive value; NPV, Negative predictive value; PLR, Positive likelihood ratio; NLR, Negative likelihood ratio; AUC, Area under the curve; uTFMP, Urinary total fluorescent metabolome profiles.

**Table 3:** Comparison of state-of-the-art non-invasive diagnostic techniques for EC.

Study	Method	Sample Size	Biomarkers	AUC	Reference
Our Study (2024)	Fluorescence spectroscopy + ML (urine)	77 EC, 96 controls, 23 benign	Spectral features	0.90	-
Meza Ramirez et al. (2022)	Infrared spectroscopy + ML (catheterized urine)	109 EC, 110 controls	Spectral features	0.99	[18]
Bouziyane et al. (2021)	RT-qPCR (tissue)	71 tumors, 53 adjacent, 54 benign	miR-21	0.93	[60]
Njoku et al. (2022)	Urine biomarkers + TSV	153 symptomatic patients	CA125 + endometrial thickness	0.96	[59]
Barr et al. (2022)	Blood biomarkers	755 patients (397 EC)	HE4, CA125	0.77	[54]

## Data Availability

The data presented in this study are available upon reasonable request from the first author. The data are not publicly available due to ethical restrictions and patient confidentiality.
